# Transcriptome Mechanism of Utilizing Corn Steep Liquor as the Sole Nitrogen Resource for Lipid and DHA Biosynthesis in Marine Oleaginous Protist *Aurantiochytrium* sp.

**DOI:** 10.3390/biom9110695

**Published:** 2019-11-04

**Authors:** Dong-Sheng Wang, Xin-Jun Yu, Xiao-Yu Zhu, Zhao Wang, Hui-Juan Li, Zhi-Peng Wang

**Affiliations:** 1 Institute of Biological Resources, Jiangxi Academy of Sciences, Nanchang 330096, China; w_d_sh@126.com; 2 Key Laboratory of Bioorganic Synthesis of Zhejiang Province, College of Biotechnology and Bioengineering, Zhejiang University of Technology, No. 18, Chaowang Road, Hangzhou 310014, China; xyzhu19@126.com (X.-Y.Z.); hzwz2019@163.com (Z.W.); 3 Department of Bioengineering, College of Chemical and Environmental Engineering, Shandong University of Science and Technology, Qingdao 266590, China; lihuijuan611@126.com; 4 Marine Science and Engineering College, Qingdao Agricultural University, Qingdao 266109, China; wangzp@ysfri.ac.cn

**Keywords:** *Aurantiochytrium* sp., nitrogen-deficiency, RNA-seq, corn steep liquor (CSL), signal transduction, DHA

## Abstract

In the current study, corn steep liquor (CSL) is evaluated as an ideal raw agro-material for efficient lipid and docosahexaenoic acid DHA production by *Aurantiochytrium* sp. Low CSL level in medium (nitrogen deficiency) stimulated the biosynthesis of lipids and DHA while inhibiting cellular growth. The transcriptomic profiles of the *Aurantiochytrium* sp. cells are analyzed and compared when cultured under high (H group), normal (N group), and low (L group) levels of CSL in the medium. The discriminated transcriptomic profiles from the three groups indicates that changes in CSL level in medium result in a global change in transcriptome of *Aurantiochytrium* sp. The overall de novo assembly of cDNA sequence data generated 61,163 unigenes, and 18,129 of them were annotated in at least one database. A total of 5105 differently expressed (DE) genes were found in the N group versus the H group, with 2218 downregulated and 2887 upregulated. A total of 3625 DE genes were found in the N group versus the L group, with 1904 downregulated and 1721 upregulated. The analysis and categorization of the DE genes indicates that the regulation mechanism of CSL involved in the perception and transduction of the limited nitrogen signal, the interactions between the transcription factors (TFs) and multiple downstream genes, and the variations in downstream genes and metabolites, in sequence, are illuminated for the first time in the current study.

## 1. Introduction

Corn steep liquor (CSL) is a major by-product of the corn wet milling industry. Because it is rich in organic nitrogen, it has been used as a crucial nitrogen resource in microbial fermentation of products such as amino acids, polypeptides, and proteins. Moreover, CSL has also been utilized in the fermentation process as a nutritional and functional supplement due to its high content of vitamins, which make a significant contribution to nutrition. Otherwise, CSL has a much lower price as a bulk agro-industrial by-product than traditional nitrogen resources such as yeast extract and peptone, which constitute the main cost in microbial fermentations. Thus, CSL has been used as a potential nitrogen resource in the microbial fermentations of various organic acids and other products [1,2].

Docosahexaenoic acid (DHA, C22:6) belongs to the n-3 polyunsaturated fatty acid (PUFA) and has received worldwide attention due to its multiple beneficial physiological functions for humans, such as improving the developments of nerves and retinas, reducing the risks of atherosclerosis, hypertriglyceridemia, hypertension, schizophrenia, and cancers, and so on [3]. Thus, DHA serves as a multi-function supplement and has been widely applied in the food, medical, and feed industries. *Aurantiochytrium* sp. is a marine protist and typical of oleaginous microorganisms, and can accumulate 50–70% dry cell weight (DCW) as lipids rich in PUFAs, such as DHA. Moreover, *Aurantiochytrium* sp. is a heterotrophic microorganism and can effectively utilize a variety of cheap substrates for fast and high-density cellular growth. Thus, *Aurantiochytrium* sp. serves as an industrial DHA-producing strain, and the DHA extracted from this microorganism has been considered to be an alternative DHA resource that can solve the problems caused by extracting traditional fish oil, such as overexploitation, environmental pollution, unpleasant odor, and so on [4]. However, the cost of producing DHA by *Aurantiochytrium* sp. that is mainly composed of nitrogen in a fermentative medium is still high, leading to a severe limitation on marketization development. Thus, various cheap nitrogen resources such as NaNO_3_ [5], alanine mother liquor [6], algae-residue [7], defatted silkworm pupa hydrolyzate [8], soybean meal hydrolysate [9], casitone [10], and so on have been used as nitrogen sources, replacing yeast extract and peptone for DHA production in *Aurantiochytrium* sp. However, CSL has never been reported as having been utilized for microbial DHA production.

Moreover, nitrogen source plays a crucial role in the regulations of cellular growth and lipid and DHA biosynthesis in *Aurantiochytrium* sp. It is well known that a limited-nitrogen condition induces lipid and DHA synthesis while inhibiting cellular growth in *Aurantiochytrium* sp. [11,12]. The mechanism of nitrogen regulation (mainly yeast extract and peptone) has been preliminarily illuminated. Jiang et al. revealed by analyzing the activities of isocitrate dehydrogenase (ICDH) and malic enzyme (ME) that nitrogen exhaustion facilitates lipid and DHA accumulation in *Aurantiochytrium* sp., which provides the NADPH for fatty acid synthesis [12]. Metabolomic technology and enzymatic activity analysis have been applied to illuminate variations in metabolites and activities of critical enzymes in the metabolic pathways of *Aurantiochytrium* sp. under different nitrogen conditions [13]. However, the transcriptomic mechanism of nitrogen regulation at the global-cell level of *Aurantiochytrium* sp. has never been reported.

Transcriptomics based on RNA-seq technology is a powerful strategy that can be used to uncover the global variations in gene expressions for responses of microbial cells to various environmental stresses. This technology has been applied to illuminate the mechanisms of various environmental stresses such as low temperature, low oxygen supplementation, and high salinity, which induce DHA biosynthesis in *Aurantiochytrium* sp. [14,15,16]. Numerous differently expressing (DE) genes as well as cellular metabolic pathways have been explored by exposing *Aurantiochytrium* sp. cells to diverse environmental stresses and monitoring DHA production [14,15].

In the current study, the availability of CSL will first be evaluated as the sole nitrogen resource for DHA production by *Aurantiochytrium* sp. Then, the transcriptomic profiles of *Aurantiochytrium* sp. cells cultured under three levels of CSL in the medium (representing different levels of nitrogen) will be analyzed and compared to illuminate the regulative mechanism of CSL as the sole nitrogen for efficient DHA production. Through this research, a global regulative model of CSL on cellular growth and lipid and DHA production in *Aurantiochytrium* sp. is put forward for the first time. This study provides a foundation for utilizing CSL to produce DHA in oleaginous microorganisms at an industrial scale. Furthermore, our research improves and enriches theories of synthesis and regulation for lipids and DHA in oleaginous microorganisms, and supports various potential targets of genetic modifications for improving lipid and DHA productivity.

## 2. Material and Methods

### 2.1. Materials

The nonadecanoic acid methyl ester was the internal standard, and was purchased from Sigma-Aldrich (St. Louis, MO, USA). BF_3_-methanol was purchased from ANPEL Laboratory Technologies Inc. (Shanghai, China). CSL was kindly supplied by the Baimai Green Biological Energy Co., Ltd. (Huaian, China). All other chemicals and reagents were purchased from Aladdin Industrial Inc. (Shanghai, China).

### 2.2. Strain and Culture Conditions

*Aurantiochytrium* sp. YLH70 was isolated from the Mangrove ecosystem in Yueqing Bay (Zhejiang, China) and was used in this study [11,17]. The *Aurantiochytrium* sp. strain was reserved at −80 °C and transferred onto the seed medium plate for activating the strain. A single strain colony was then inoculated into the seed medium and cultured at 28 °C and 150 rpm for 24 h. The seed culture was then transferred into the CSL media containing three levels of CSL and cultured at 28 °C for 120 h. The seed medium was composed of 20 g/L glucose, 10 g/L CSL and 20 g/L sea salt. The carbon resource and salt in CSL medium was composed of 60 g/L glucose and 20 g/L sea salt, and the effects of different concentrations of CSL (0.5 g/L, 1g/L, 5 g/L, 10 g/L, 20 g/L and 30 g/L) in the CSL medium on biomass, lipid and DHA production in *Aurantiochytrium* sp. were evaluated. For transcriptomic analysis, three levels of CSL in medium, including were set, including 20, 5 and 1 g/L representing high (H group), normal (N group) and low (L group) levels of nitrogen in medium, respectively. Each group had three replications.

### 2.3. Biomass Determination

The *Aurantiochytrium* sp. biomass was determined in terms of dry cell weight (DCW), and 50 mL samples of cell suspensions were centrifuged at 4 °C and 10,000× *g* for 5 min after washing twice with distilled water. The cell pellets were lyophilized to a constant weight at −50 °C for approximately 48 h.

### 2.4. Lipid and Fatty Acids Analysis

Lipid and fatty acids were analyzed based on the methods in our previous studies [18]. The lyophilized cell was first ground into a fine powder using a mortar and pestle under liquid nitrogen, then extracted with chloroform/methanol (2:1, *v*/*v*) at room temperature. The lipid extract was dried to a constant weight by evaporation. Afterward, fatty acid methyl esters (FAMEs) were prepared based on our previous method [11].

### 2.5. Sample Collection and Pretreatment

The *Aurantiochytrium* sp. cells cultured in CSL media with three levels of CSL (the H, N and L groups) were each sampled for 96 h, respectively. A volume of 10 mL cell samples from the three groups were centrifuged at 5000 rpm at 4 °C for 5 min. The pellets were then washed twice using icy deionized water, immersed in RNAlock Reagent, and preserved at − 80 °C for use.

### 2.6. RNA Isolation

Total RNA was extracted from the *Aurantiochytrium* sp. samples cultured in the media containing three levels of CSL (H, N and L groups), respectively. Total RNA was extracted using Invitrogen TRIzol Reagent (Thermo Fisher Scientific, Waltham, MA, USA) based on the manufacturer’s protocol. The RNA purity and integrity were analyzed by Nanodrop (Thermo Fisher Scientific, Waltham, MA, USA) and Agilent2100 Bioanalyzer (Agilent Technologies, Palo Alto, CA, USA), respectively.

### 2.7. Library Preparation and Sequencing

The library for next-generation sequencing (NGS) was prepared based on the manufacturer’s protocol (NEBNext Ultra Directional RNA Library Prep Kit for Illumina). mRNA was fragmented into short fragments by mixing with the fragmentation buffer. Then, reverse transcriptase and random hexamer primers were used to synthesize the first cDNA strands of the fragmented mRNA. The second-strand cDNA was synthesized using Second Strand Synthesis Enzyme Mix, including buffer, dNTPs, RNase H, and DNA polymerase I. Afterwards, sequencing adapters were ligated to the short cDNA fragments. The cDNA with adapter was purified by agarose gel electrophoresis, and the suitable fragments were selected as templates for PCR amplification. The PCR products were cleaned up using AxyPrep Mag PCR Clean-up (Axygen), validated using an Agilent 2100 Bioanalyzer (Agilent Technologies, Palo Alto, CA, USA), and quantified by Qubit 2.0 Fluorometer (Invitrogen, Carlsbad, CA, USA). Finally, the RNA-seq library was sequenced using an Illumina HiSeq system based on the manufacturer’s instructions (Illumina, San Diego, CA, USA).

### 2.8. RNA-Seq Data Processing and Transcriptome Analysis

The optimized Fastq-clean pipeline was used to clean the raw reads from the Illumina system. The low-quality sequences on both ends of the raw reads with a Q20 value more than 10% were removed. Then, the adapter and PCR primer sequences from the 3′-end of the reads were trimmed to produce the cleaned reads with lengths more than 75 bp [19]. Quality control was performed for every sample to make sure it met the requirements.

The clean reads were filtered from raw data and assembled into contiguous sequences (contigs) by Trinity software, combining the Inchworm, Chrysalis, and Butterfly modules [20]. The abundances of transcripts were normalized as reads per kilobase of eonmodel per million mapped reads (RSEM, v1.2.6) to estimate the gene expression levels of each sample [21]. The differential expression (DE) genes among three groups of samples were analyzed using the DESeq Bioconductor package. The expression levels of unigenes were based on the mean value of the three samples as the level of this group and compared among the three groups. The model was based on the negative binomial distribution. The p-value was adjusted by Benjamini and Hochberg’s method of controlling the false discovery rate (FDR). A *p*-value less than 0.05 was set to detect the differentially expressed (DE) genes [22].

For functional enrichment, the DE genes were mapped to terms in Gene Ontology (GO) and KEGG (Kyoto Gene and Genomic Encyclopedia) databases (http://www.geneo ntology.org and http://en.wikipedia.org/wiki/KEGG). The GOseq R package for the DE genes was used to conduct GO enrichment analysis, which annotated a list of enriched genes with significant *p*-values less than 0.05 and absolute values of log_2_FoldChange more than 1. The KOBAS software was used to enrich the DE genes in the KEGG pathway [23].

### 2.9. Quantitative Real-Time Reverse Transcription PCR (qRT-PCR)

The expression levels of the eight specific DE genes were validated through qRT-PCR. The gene-specific primer sequences were designed by Primer Premier 5.0 and listed in Table S1. qRT-PCR was performed in the CFX96 Touch qRT-PCR system (BIO-RAD, Hercules, CA, USA). The PCR procedure and calculations of relative expression values were based on our previous method [22]. The level was based on the mean value of the three samples as the level of this group and compared among the three groups.

### 2.10. Metabolomic Validation

The metabolomics procedure, including sample collection and pretreatment, derivation, GC-MS analysis and data processing and analysis, were performed based on our previous methods [18]. One-way analysis of variance (ANOVA) *t*-tests were performed using SPSS 12.0 (New York, NY, USA) software to analyze the distribution of the concentrations and logarithmically transformed levels for metabolites in this study. The level was based on the mean value of the three samples as the level of this group and compared among the three groups.

### 2.11. Enzymatic Validation

Several detectable enzymes responding to the DE genes were analyzed to validate the transcriptomic data. The activities of malic enzyme (ME), glucose-6-phosphate dehydrogenase (G6PD), isocitrate dehydrogenase (IDCH), and protease were investigated using previously described methods [22,24]. The protein concentration was determined using the Bradford method with bovine serum albumin (BSA) as a standard. The level was based on the mean value of the three samples as the level of this group and compared among the three groups.

## 3. Results

### 3.1. Evaluation of CSL for Lipid and DHA Production by Aurantiochytrium sp.

**Figure 1:**
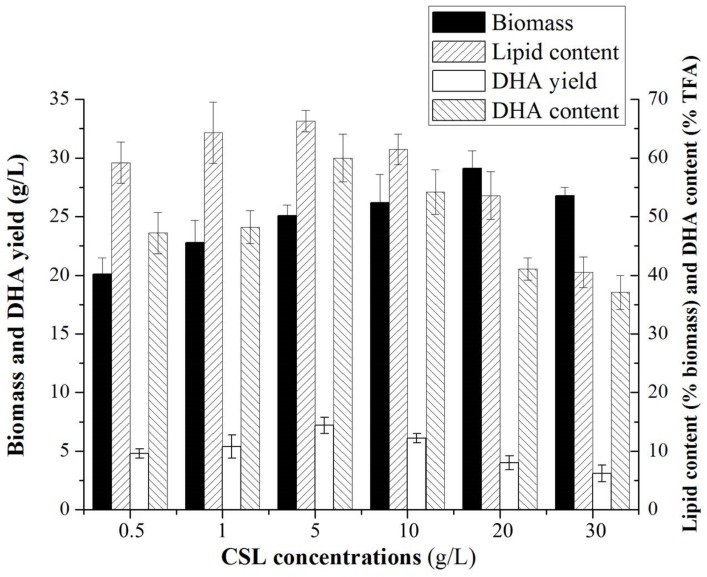
Effects of different CSL-levels in medium on biomass, lipid, and DHA yields in *Aurantiochytrium* sp. cells. All data are the means of three replicates; vertical bars represent error bars with values equal to the standard error of the mean.

In the current study, availability of utilizing CSL as a sole nitrogen source for lipid and DHA production by *Aurantiochytrium* sp. was evaluated. As shown in Figure 1, the lipid content, DHA yield and content reached the highest level (66.3% of biomass, 7.2 g/L and 66% of TFA) when the concentration of CSL reached to 5 g/L with a C/N value of up to 12. However, the optimal biomass (29.1 g/L) was obtained when the concentration of CSL was 20 g/L with a C/N value of 3. Moreover, the results from Figure 1 show that the effects of CSL on biomass, lipid content, DHA yield and content in *Aurantiochytrium* sp. were regular and significant, and that CSL as a sole nitrogen source plays a crucial role in regulation of lipid and DHA biosynthesis in *Aurantiochytrium* sp. On the other hand, the transcriptomic mechanism of CSL for biomass, lipid and DHA biosynthesis in *Aurantiochytrium* sp. is still unclear. Thus, based on the results in Figure 1, the transcriptome profiles of the samples from the media containing 5, 10 and 20 g/L of CSL, representing the low (L group, high lipid and DHA levels), normal (N group) and high (H group, high biomass level) levels of nitrogen were further analyzed to illuminate the regulation mechanism of CSL on biomass, lipid, and DHA biosynthesis in *Aurantiochytrium* sp.

### 3.2. Illumina Sequencing, Reads Assembly, and Functional Annotation

**Figure 2:**
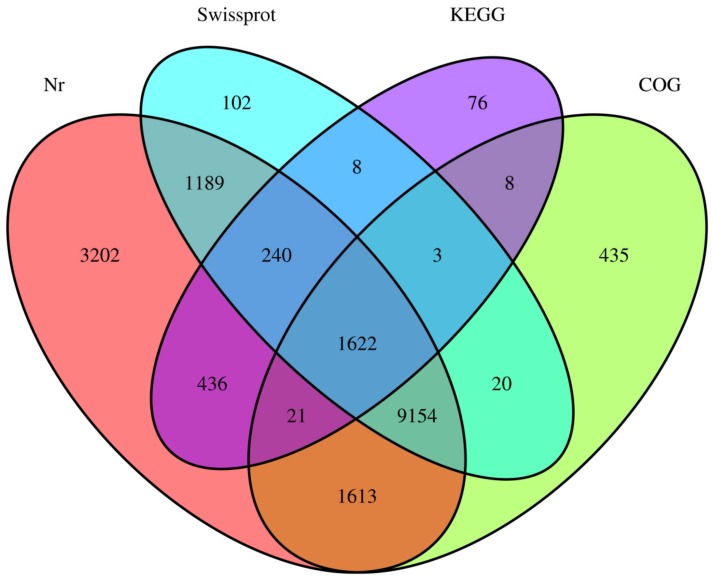
The distribution of unigene annotation in four databases.

**Figure 3:**
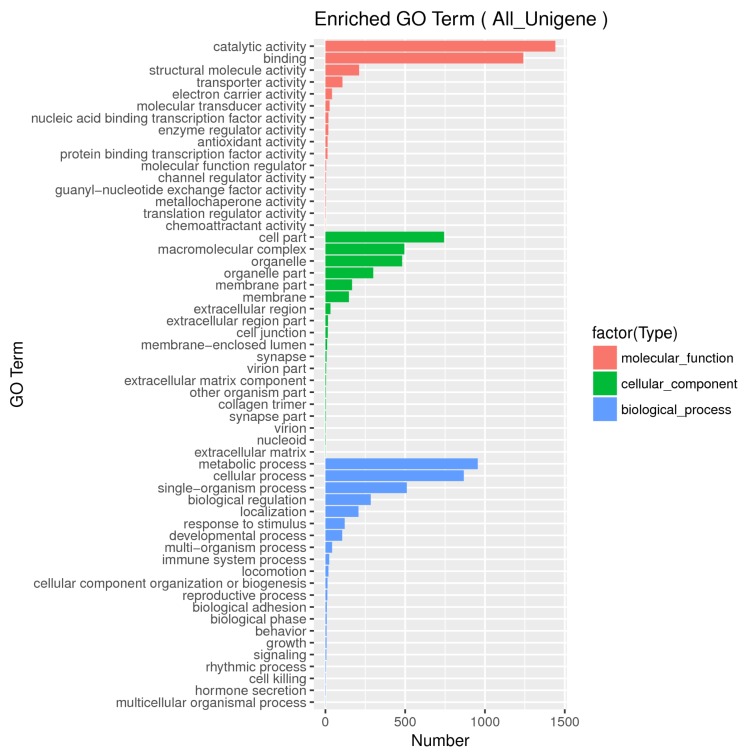
Categorization of GO function for unigenes in *Aurantiochytrium* sp. cells.

**Figure 4:**
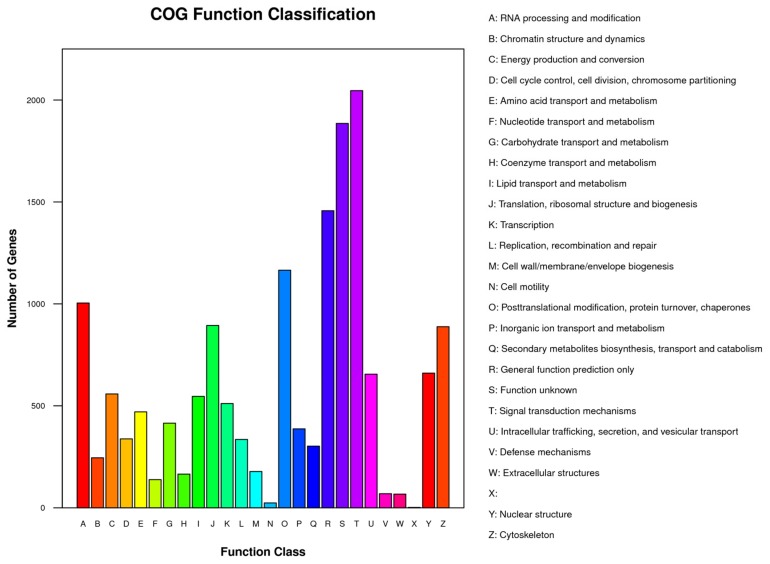
COG function classification for unigenes in *Aurantiochytrium* sp. cells.

After pretreatments of transcriptome data for quality control, approximately 40.5–57.5 million clean reads with a Q20 value range of 96.7–97.2% among the three sample groups were obtained (Table S2). These cleaned reads were further assembled into 61,163 contigs (unigenes) using Trinity. A range length of 201–33,200 bp, a mean length of 1347.9 bp and a N50 length of 3399 bp were found in these unigenes (Table S3). Using the Blast programing, the total number of sequences out of 61,163 unigens having at least one blast hit against four databases (NR, SwissProt, KEGG and COG) was 18,129 (29.5%), and the distributions of these mapped unigenes are summarized in Table 1 and Figure 2. The numbers of the unigenes annotated in the Nr, SwissProt, KEGG and COG were 17477, 12338, 2414, and 12876 (data not shown). The distributions of identity value in the Nr and SwissProt databases, presenting the percentage distributions of the mapped unigenes covered by the best hits, are shown in Figures S1 and S2. The unigenes mapped in the Nr database were further annotated by Gene Ontology categories (GO terms) at 56 levels in three domains: molecular function (MF), cellular component (CC), and biological process (BP). The total numbers of unigenes re-annotated by GO terms were 3148, 2438, and 3204 for MF, CC, and BP, respectively (Figure 3). All of the unigenes were further mapped to the COG database, and 15,404 of them were clustered into 26 functional COG groups (Figure 4). Only 546 unigenes (3.54%) were clustered into the “lipid transport and metabolism” category, indicating that lipid and DHA synthesis in *Aurantiochytrium* sp. depends not only on lipid metabolism, but also on other physiological functions such as signal transduction, posttranslational modification, and so on (Figure 4).

### 3.3. Transcriptome Profiles of Aurantiochytrium sp. Cells under Three-Level CSL Conditions

There were three sample groups (H, N, and L), and thus two comparison groups—the N versus H group, and the N versus L group—were constructed, and the transcriptome profiles of them were analyzed. A total of 5105 DE genes, with 2218 downregulated and 2887 upregulated, were found in the N versus H group, and a total of 3625 DE genes, with 1904 downregulated and 1721 upregulated, were found in the N versus L group (Figure S3). The PCA analysis discriminated the H group and the N–L group at component 1, and the N–L group was further discriminated at component 2 (Figure S4). The DE gene profiles from the three sample groups were also clearly distinguished by HCA analysis (Figure S5). These results suggest that CSL levels in medium indeed significantly disturb the expression of genes at a global cellular level. The DE genes were re-annotated by GO term at 56 degrees in three GO domains (Figure S6). Meanwhile, a total of 2137 DE genes from the N versus H group and 1473 DE genes from the N versus L group were clustered into 26 functional KOG groups, with most of them categorized “signal transduction mechanisms” or “general function prediction only” (Figure S7).

### 3.4. DE Genes Related to the CSL Regulation of Growth and Lipid and DHA Synthesis

**Figure 5:**
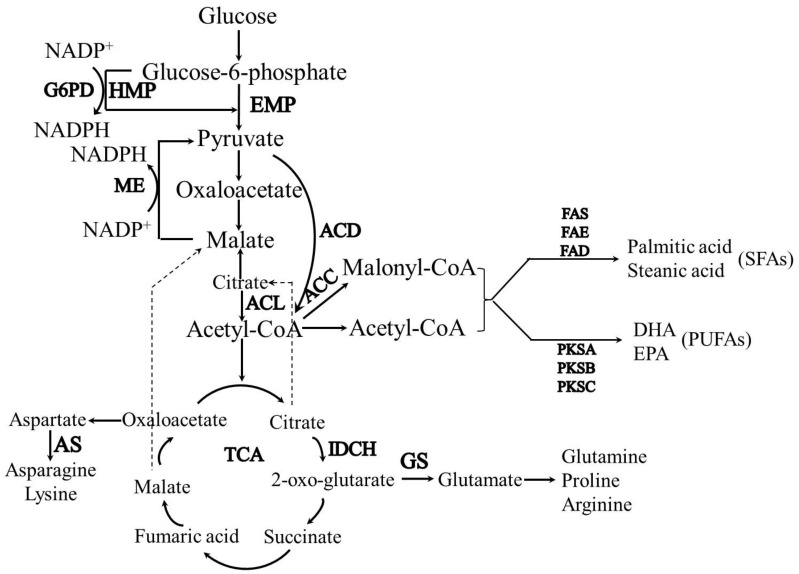
The metabolic network and key enzymes in *Aurantiochytrium* sp. cells. G6PD, glucose-6-phosphate dehydrogenase; ME, malic enzyme; ACL, ATP citrate lyase; ACC, Acetyl-CoA carboxylase; FAS, fatty acid synthase; FAE, fatty acid elongase; FAD, fatty acid desaturase; PKS, polyketide synthase; IDCH, Isocitrate dehydrogenase; AS, Asparagine synthase; GS, Glutamate synthase; EMP, glycolytic pathway; HMP, Hexose Monophosphate Pathway; TCA, Tricarboxylic acid cycle.

As described above, the regulation of CSL as a nitrogen source on biomass, lipid, and DHA synthesis involve various pathways at a whole-cell level. Thus, the DE genes identified from the three sample groups were further analyzed and categorized into “fatty acid synthesis”, “central carbon metabolism”, “nitrogen metabolism” and “signal transduction”, which are closely related to biomass, lipid, and DHA synthesis (Table 1). In the “fatty acid synthesis” category, the expression levels of the polyketide synthase (PKS) subunits A, B, and C (which functioned in combination to synthesize PUFAs through the PKS pathway in *Aurantiochytrium* sp.) were increased as the level of CSL in medium decreased. Oppositely, the expression levels of the fatty acids synthase, elongase, and desaturase, which catalyze the synthesis of saturated fatty acids (SFAs) through the FAS pathway in *Aurantiochytrium* sp., were decreased as the level of CSL in medium decreased. A metabolomics analysis (Table S4) showed that the contents of palmitic acid and stearic acid, the two primary SFAs in *Aurantiochytrium* sp., were positively correlated with the CSL level in medium, while the contents of docosapentaenoic acid and docosahexaenoic acid, the two primary PUFAs in *Aurantiochytrium* sp., were negatively correlated with the CSL level in medium. These results validated the transcriptomic results and suggested that the limited-nitrogen condition could facilitate PUFA synthesis and inhibit the SFA synthesis. In the “central carbon metabolism” group, shown in Table 1 and Figure 5, the expressions of two DE genes—encoding acetyl-CoA carboxylase (ACC) and acyl-CoA synthetase (ACS)—that catalyzed acetyl-CoA and acetate to produce malonyl-CoA and acetyl-CoA (which are the carbon skeleton for fatty acid synthesis), were upregulated under a low CSL level in medium (L group). Meanwhile, the expression profile of glucose-6-phosphate dehydrogenase (G6PD), which is responsible for producing reducing power NADPH for fatty acid synthesis, was significantly induced by the low-level CSL sample (L group). These results indicate that either the low CSL level or nitrogen-deficiency conditions induced fatty acid synthesis by enforcing a carbon skeleton and reducing power supplementation.

**Figure 6:**
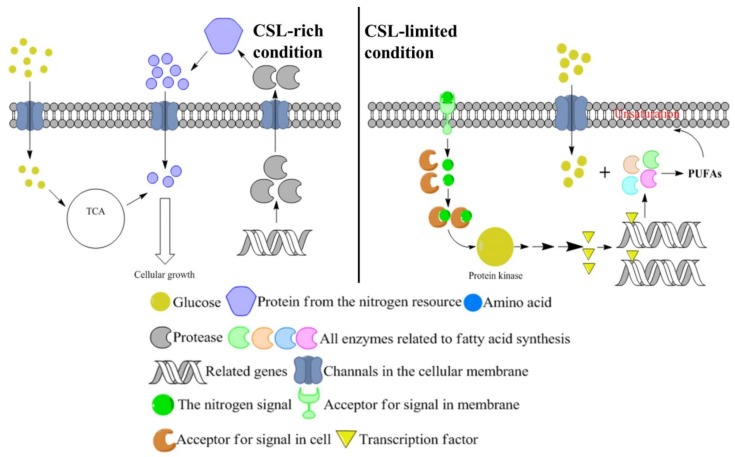
The regulation model in *Aurantiochytrium* sp. cells under the CSL-rich condition (left) and CSL-limited condition (right).

As CSL in the medium is the sole nitrogen resource, the DE genes from the three sample groups involved in the “nitrogen metabolism” are identified and analyzed in the current study. The expression levels of two DE genes, glutamate synthase and asparagine synthase (which catalyze α-ketoglutarate and oxaloacetate from the TCA to produce glutamate and asparagine, respectively (Figure 5)), increased as the CSL level increased in medium (Table 1). A metabolomic analysis was applied to validate the transcriptomic data. As shown in Table S4, the contents of glutamate and asparagine (the direct products catalyzed by the glutamate synthase and asparagine synthase), increased as the CSL level in medium increased. Meanwhile, the proline, lysine, and citrate derived from the TCA pathway increased with increases of the CSL of each medium. These results indicate that the nitrogen-rich medium (high CSL level) could promote more carbon flux into the TCA cycle and enforce amino acid and protein synthesis in *Aurantiochytrium* sp. for cellular growth. Moreover, aspartyl protease hydrolyzed the nitrogen source in the medium into oligopeptides or amino acids, which were absorbed into the cell for biomacromolecules synthesis, and its gene expression increased under higher CSL levels in medium (Table 1). This results indicate that aspartyl protease was induced by the CSL-rich condition to produce oligopeptides or amino acids that were more available for cellular growth (Figure 6).

The CSL regulation in the cell was at a global level. Thus, signal transduction and related transcription factors (TFs) played essential roles in the global regulation of CSL in *Aurantiochytrium* sp. cells. As shown in Table 1, the genes encoding two signal transduction elements—the seine/threonine protein kinase and the Ca^2+^/calmodulin-dependent protein kinase—and a transcription factor, the MYB transcription factor, were identified as DE genes, and their expression profiles increased under decreased CSL levels. This result indicate that the low CSL level condition facilitated the specific signal pathways and transcription factors to respond to the limited-nitrogen stress (Figure 6).

### 3.5. Validation of the Transcriptomic Data by qRT-PCR

To validate the RNA-seq results, a total of seven DE genes were selected and amplified using qRT-PCR technology. As shown in Table 1, the expression patterns of the selected DE genes were consistent with those from the transcriptomic profiles, confirming the reliability of the RNA-seq results.

## 4. Discussion

In the current study, CSL is evaluated as an ideal sole nitrogen resource for cellular growth and lipid and DHA production in *Aurantiochytrium* sp. in terms of productivity, nutrition, and cost. The optimal biomass, lipid content, and DHA yield and content reached up to 29.1 g/L and 66.3% of biomass, and 7.2 g/L and 60% of TFA with the CSL as the sole nitrogen source. These results are comparable to, or even higher than, those from traditional nitrogen sources such as yeast extract and peptone [25,26]. CSL is rich in not only organic nutrients (those with carbon and nitrogen contents), but also trace nutrients (with B vitamins and metal ions) [27], which are essential for cellular growth and lipid and DHA accumulation in *Aurantiochytrium* sp. [28]. Thus, these is no need to add extra trace elements to CSL medium to enhance DHA production by *Aurantiochytrium* sp. (data not shown), which reduces the cost and complexity of DHA production. Moreover, CSL has the relatively low price of 200 USD/ton due to its wide availability, which is much less than the price of yeast extract and peptone (1000–1250 USD/ton) [29]. Thus, CSL is an ideal low-cost raw material for DHA production from *Aurantiochytrium* sp.

Nitrogen is an important element and regulator of biomass and lipid and fatty acid accumulation in oleaginous microorganisms. It is well known that limited-nitrogen (nitrogen starvation or deprivation) conditioning stimulates lipid and DHA synthesis and inhibits the cellular growth of various microorganism strains [30]. In the present study, a relatively low CSL concentration (5 g/L, C/N = 12) in medium favored lipid and DHA accumulation in *Aurantiochytrium* sp., while a relative high CSL concentration in medium (20 g/L, C/N = 3) favored cellular growth of *Aurantiochytrium* sp. (Figure 1). This is similar to previous findings. Thus, the transcriptome mechanism of CSL regulation is illuminated by the present study.

The unsaturation of fatty acids determines the biophysical characteristics of cell membranes and the proper function of membrane-attached proteins. Thus, microorganisms are able to vary their fatty acid profiles and increase their tolerances to various environmental stresses [31]. In the present study, a limited-nitrogen condition (low level of CSL in medium) increased the unsaturation degree of fatty acids by enforcing the PKS pathway for PUFA synthesis and weakening the FAS pathway for saturated fatty acid (SFA) synthesis at a transcriptional level (Table 1 and Figure 5) [32]. Meanwhile, the synthesis of carbon skeleton and reduced power for fatty acids during central carbon metabolism were enhanced at a transcriptional level under the low CSL condition (Table 1). Besides the limited-nitrogen condition, various environmental stresses, such as low-oxygen supply [15], 6-BAP treatment [22], cold stress [14], and so on were found to induce the DHA synthesis in *Aurantiochytrium* sp. Moreover, genetic modifications to various genes (e.g., encoding acetyl-CoA synthetase, malic enzyme (ME), glucose-6-phosphate dehydrogenase (G6PD), and so on), were applied to further improve DHA productivity in *Aurantiochytrium* sp. [33,34].

The metabolism of nitrogen is pivotal to CSL regulation in the synthesis of DHA in *Aurantiochytrium* sp. *Aurantiochytrium* sp. is a marine heterotrophic protist and has evolved a multi-enzyme system that plays an essential role in the material cycle of the marine ecosystem and is able to utilize various substrates for DHA production [35]. In the current study, a DE gene encoding an aspartyl protease from *Aurantiochytrium* sp. was induced as the CSL level in the medium was increased (Table 1). CSL is rich in 40% protein and 16% nitrogen free extract, making it an ideal nitrogen source for microbial fermentation [36]. Thus, our results indicate that the aspartyl protease from *Aurantiochytrium* sp. is inducible and that more proteins are available for metabolism given a higher level of CSL in medium, which explains why the high level of CSL in medium favored the growth of *Aurantiochytrium* sp. (Figure 6). In addition, glutamate and asparagine, derived from the TCA cycle, were improved with a high CSL level by enforcing their synthases’ expression. A metabolomics analysis showed that proline and lysine were also induced by a high level of CSL. This result implied that the nitrogen-rich medium could increase the synthesis of amino acids by enforcing the carbon flux into the TCA cycle for protein synthesis or cellular growth while inhibiting the fatty acid synthesis (Figure 6). In contrast, some stimulators such as gibberellin and 6-BAP have tuned down the amino acid synthesis and TCA cycle, while enforcing fatty acid and lipid synthesis in *Aurantiochytrium* sp. [18,37]. Thus, nitrogen plays an important role in balancing cellular growth and metabolite synthesis in *Aurantiochytrium* sp. through multi-target regulations.

Signal transduction and transcription factors (TFs) are the pivotal linkage between the environmental stress and the downstream regulations of genes and metabolites. In the current study, two signal transduction elements—the serine/threonine protein kinase (MAPK), and the Ca^2+^/calmodulin-dependent protein kinase—and one transcription factor, MYB, were induced significantly under the limited-nitrogen condition (Table 1). Moreover, a number of environmental or chemical stimuli, such as low temperature and 6-BAP treatments, were also found to induce signal transduction systems to enhance lipid and DHA productions in *Aurantiochytrium* sp. [22,38]. The signal transduction system has been proven to play an essential role in microbial responses to various biotic and abiotic stresses [39]. Various proteins serve as transcription factors (TFs) associating signal pathways with the downstream regulation of multi-genes by interacting with their promoter sequences. In plants, MYB TFs can activate anthocyanin-synthesis genes under a depleted-nitrogen condition to improve anthocyanin accumulation. Overexpression of the TF bHLH or mutation and attenuation of TF Zn(II)_2_Cys_6_ in the *Nannochloropsis* genus has enhanced their growth rate, nutrient uptake, and FAME productivity under normal and stressed conditions [40]. These results determine that the TFs serve as critical targets for genetic modification to enhance lipid and DHA productivity under various stressed conditions.

## 5. Conclusions

In the current study, the CSL was proven to be an ideal low-cost agro-substrate for efficient lipid and DHA production by *Aurantiochytrium* sp., which could reduce the cost of DHA and increase the utilization of CSL as a bulk agro-waste. The level of CSL in medium has a significant effect on growth and lipid and DHA synthesis in *Aurantiochytrium* sp., and the transcriptomic analysis in this study illuminates a novel regulation mechanism of CSL involving the perception and transduction of a limited-nitrogen signal, interactions between the transcription factors and the multiple downstream genes, and variations in downstream genes and metabolites. This study enriches and completes the synthesis and regulation theory of microbial lipids and DHA, and provides various potential targets of genetic modifications for further productivity improvement.

## Figures and Tables

**Table 1:** Variations in the expressions of key genes in the *Aurantiochytrium* sp. cells cultured at three CSL levels. Data are given as means ± standard deviation, n = 3; CSL, corn steep liquor. * *p* < 0.05, ** *p* < 0.01.

Pathways	Gene ID	Description	FPKM			RT-PCR Validation		
			H Group	N Group	L Group	H Group	N Group	L Group
**Fatty Acid Synthesis**	**DN15169**	**Polyketide Synthase Subunit A**	66 ± 2.1	137.1 ± 3.6	211 ± 5.9	48.3 ± 2.4 **	119 ± 2.7	198 ± 3.1 *
	DN16202	**Polyketide Synthase Subunit B**	1.6 ± 0.4	3.8 ± 0.6	28.9 ± 2.1	10.8 ± 3.1 *	15.7 ± 2.1	30.4 ± 2.6 **
	DN15010	**Polyketide Synthase Subunit C**	1.6 ± 0.7	28.9 ± 2.1	32.3 ± 2.4	3.2 ± 1.9 **	20.4 ± 2.8 **	38.4 ± 2.1
	DN16221	**Fatty Acid Synthase**	43.7 ± 1.6	35.9 ± 1.6	29.5 ± 2.1	57.2 ± 3.2 **	40.2 ± 3.6	35.2 ± 4.1 **
	DN14594	**Fatty Acid Elongase**	13.4 ± 0.9	6.1 ± 0.9	3.7 ± 0.6			
	DN5965	**Fatty Acid Desaturase**	9.9 ± 1.2	9.4 ± 1.2	8.2 ± 0.7			
**Central Carbon Metabolism**	DN12414	**Glucose-6-phosphate dehydrogenase**	15.1 ± 0.9	54 ± 1.4	68.6 ± 2.6	10.5 ± 0.9 **	43.2 ± 4.2	54.2 ± 1.5 *
	DN15488	**Acyl-CoA Synthetase**	44.6 ± 2.5	47.8 ± 0.8	109.3 ± 2.6			
	DN9564	**Acetyl-CoA Carboxylase**	9.1 ± 2.1	20.3 ± 1.4	23.6 ± 1.2			
**Nitrogen Metabolism**	DN12917	**Glutamate Synthase**	33.7 ± 2.1	14.8 ± 1.5	12.5 ± 0.5			
	DN12820	**Aspartyl Protease**	8.7 ± 0.7	7.1 ± 0.9	2.4 ± 0.5			
	DN14856	**Asparagine Synthase**	2.5 ± 0.3	1 ± 0.5	0.8 ± 0.5			
**Signal Transduction**	DN11343	**MYB Transcription Factor**	16 ± 0.9	19.6 ± 1.8	23.5 ± 2.1	12.5 ± 2.7 *	20.5 ± 3.1	28.4 ± 1.8 *
	DN15563	**Serine/Threonine Protein Kinase**	10.6 ± 1.4	11.1 ± 0.9	31.5 ± 2.6			
	DN11433	**Ca2+/Calmodulin-Dependent Protein Kinase**	1.1 ± 0.5	9.6 ± 0.7	10.5 ± 2.1	0.7 ± 0.2 **	5.3 ± 0.8	12.4 ± 1.7 **

## Supplementary Materials

The supplementary materials are available online at https://www.mdpi.com/2218-273X/9/11/695/s1.
