# Supplementary material for: Transcriptome Mechanism of Utilizing Corn Steep Liquor as the Sole Nitrogen Resource for Lipid and DHA Biosynthesis in Marine Oleaginous Protist Aurantiochytrium sp
Source: Biomolecules. 2019 Nov 4;9(11):695. doi: 10.3390/biom9110695 (PMC6920895; doi:10.3390/biom9110695)
Supplement: Supplementary file 1 [file biomolecules-09-00695-s001.pdf]

Table 1. Primers for genes validated by Quantitative real-time PCR (qRT-PCR).

Genes	Primers
Fatty acid synthase	F-GTTAAATCGAGTTTCAGTTGATTTTCT<br>R-TTCAGTATGACTATTGAAAGGAGCG
Polyketide synthase subunit A	F-GAGCCCGCCGAAATCCT<br>R-TGCCCTGCGAAGTGAAT
Acetyl-CoA carboxylase	F-GGCTGGCTCCTTTGGTA<br>R-GTTGATGCGGAAGTGGT
Glucose-6-phosphate dehydrogenase	F-CCAGAGACTCCTTCGGCATAT<br>R-CCTCCAACACCTCGTCAAGA
Acetyl-CoA synthetase	F-TCAAGAAAGATAGCAAGCATGCA<br>R-TTTGGCTTGCCTCGCTC
MYB transcription factor	F-ATGCGGTTCTCCCTTATGG<br>R-TTAAACATACCGTGCAAAGAGC
Ca^{2+}/calmodulin-dependent protein kinase	F-GAAAGAAAGAAAGAAAGAAAGAAAG<br>R-AAAGTTATAAAATGCATTTTTGATT
18SrDNA	F-TCCTGCCAGTAGTCATATGCTCG<br>R-GTTCGTCTTTCGGAAATCCAAG

Table S2 Quality control of data after processing

Sample	Length	Reads	Bases	Q20 (%)	GC (%)
H1	148.6	42989792	6386281950	97.2	51.2
H2	148.8	45483646	6767315487	97.2	51.2
H3	148.8	57519590	8558929008	97.3	51.4
L1	148.8	42977010	6395670151	96.8	51.5
L2	148.9	43086034	6414994149	97	51.4
L3	148.9	42801718	6372728869	96.7	51.9
N1	148.9	40549738	6038730515	96.8	51.9
N2	148.9	53000360	7890422993	96.7	51.7
N3	148.7	43539702	6472475247	96.9	52.4

H, the high-level corn steep liquor (CSL) group; L, the low-level CSL group; N, the normal-level CSL group.

Table S3 Number, base composition and length of unigenes in *Aurantiochytrium* sp.

Type	Sequences	Bases	Min	Max	Average	N50
All Unigenes	61163	82442661	201	33200	1347.9	3399

Table S4 The relative contents of key metabolites in the *Aurantiochytrium* sp. cells from three CSL-level groups. Data are given as means ± standard deviation, n = 3; CSL, corn steep liquor.

Metabolites	Relative content in cell		
	H group	N group	L group
glutamate	9.3±1.2	7.9±1.5	3.5±0.5
asparagine	6.4±0.5	4.5±0.9	3.5±1.2
proline	4.2±0.3	3.2±0.7	1.4±0.4
lysine	5.3±0.7	4.7±1.5	3.2±0.7
citrate	3.8±1.0	2.9±1.5	0.9±0.3
Palmitic acid	8.2±1.2	6.3±0.7	5.3±1.2
Stearic acid	6.2±1.0	3.2±0.6	1.4±0.5
Docosapentaenoic acid	2.1±0.4	4.3±0.7	7.3±1.2
Docosahexaenoic acid	6.2±1.0	8.3±0.9	12.3±1.3

Table S5 The activities of three NADPH-producing enzymes under three CSL levels conditions. Data are given as means ± standard deviation, n = 3; CSL, corn steep liquor.

Enzymes	Activities		
	H group	N group	L group
glucose-6-phosphate dehydrogenase	3.2±0.7	5.8±1.2	9.3±1.2
malic enzyme	2.1±0.9	3.7±1.4	6.3±1.2
isocitrate dehydrogenase	0.9±0.1	3.4±0.8	5.3±0.9

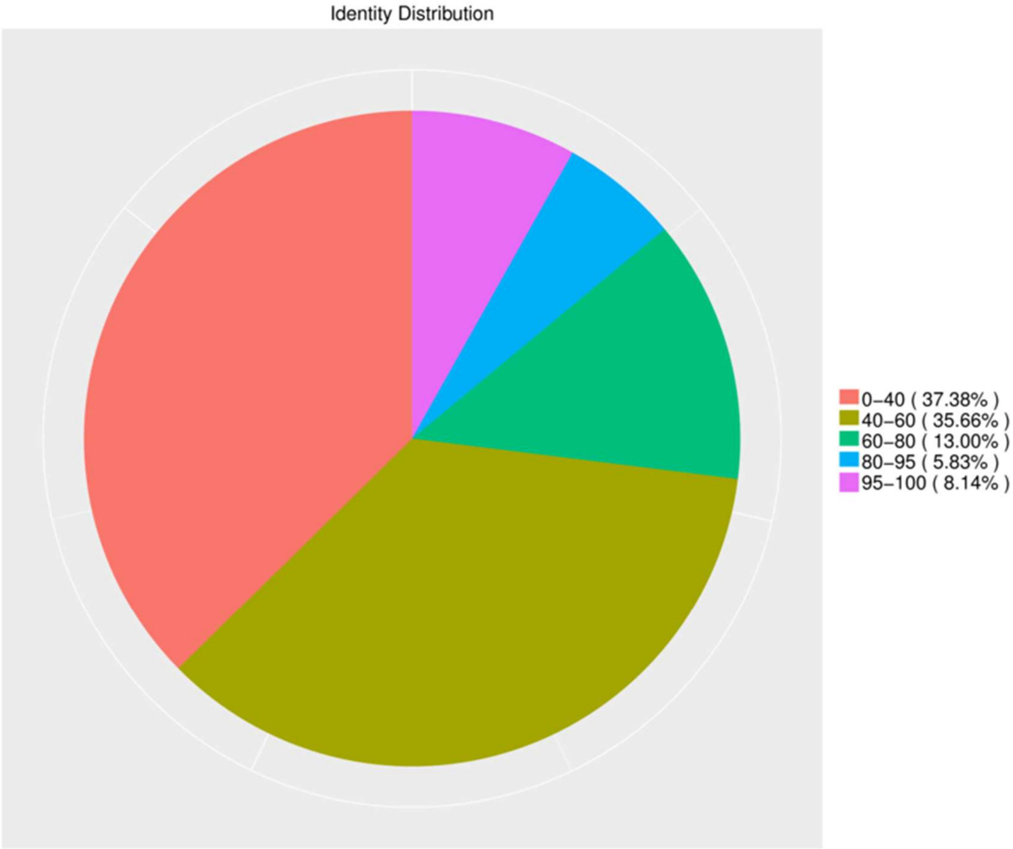

Fig.S1 Identity distribution in the Nr database.

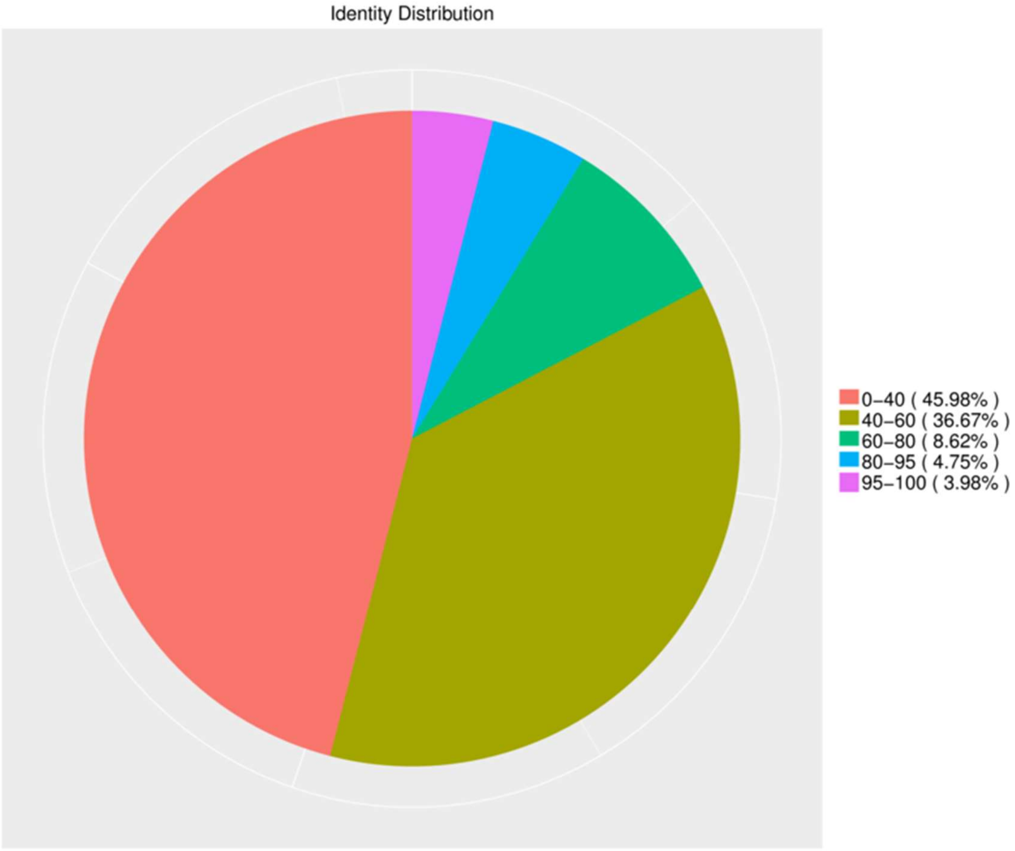

Fig.S2 Identity distribution in the SwissProt database

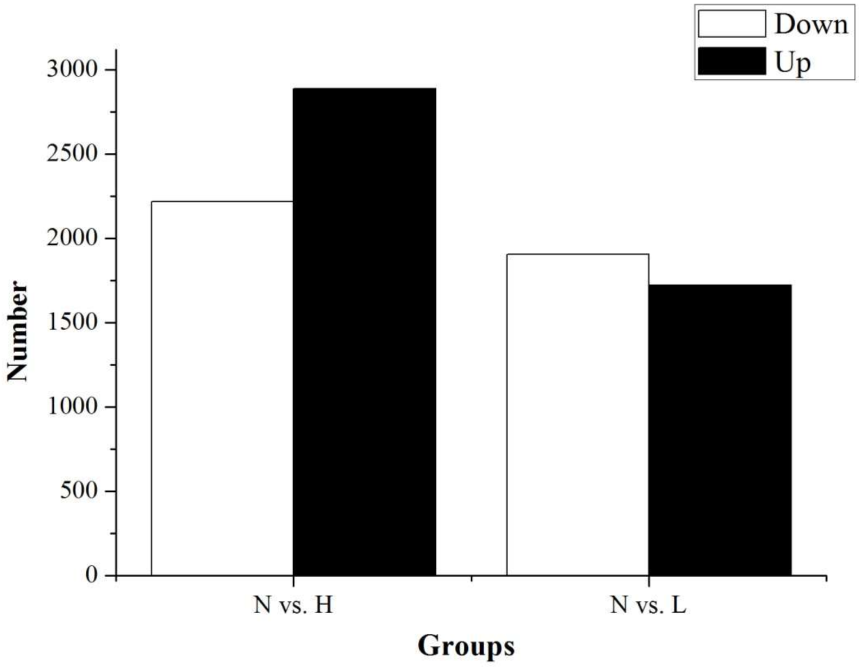

Fig. S3

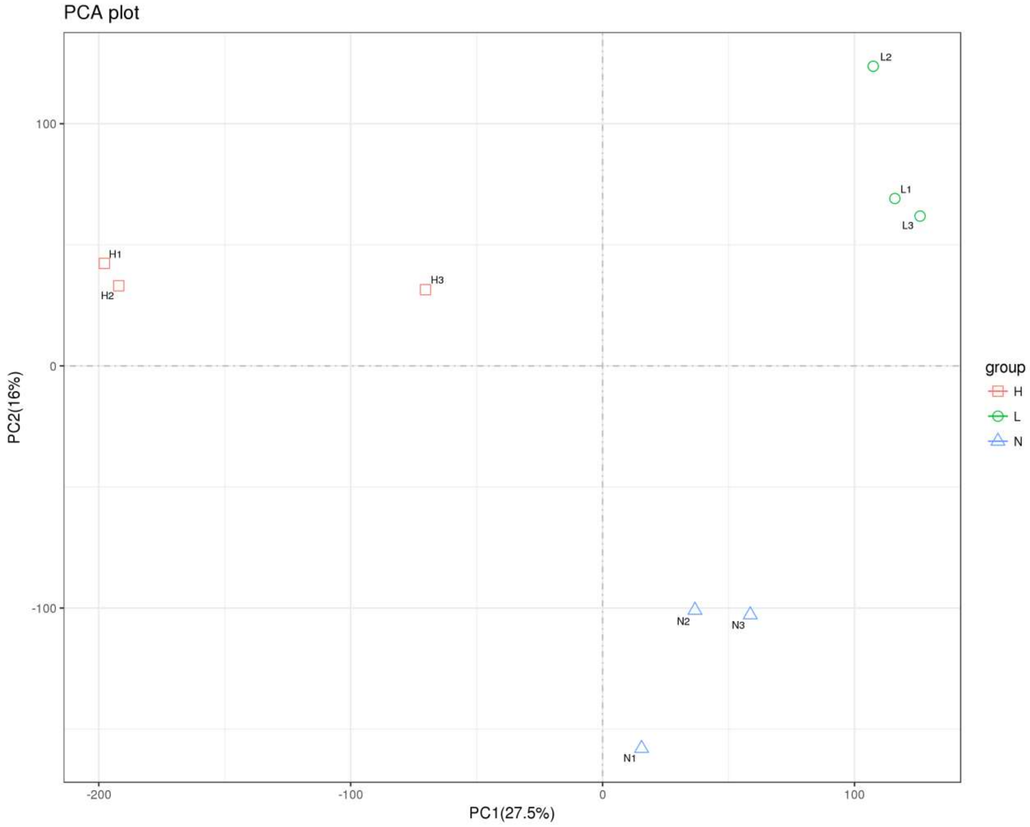

Fig.S4 PCA analysis of the DE genes from the three different groups of *Aurantiochytrium* sp. cells.

H: the high CSL-level group; N, the normal CSL-level group; L, the low CSL-level group.

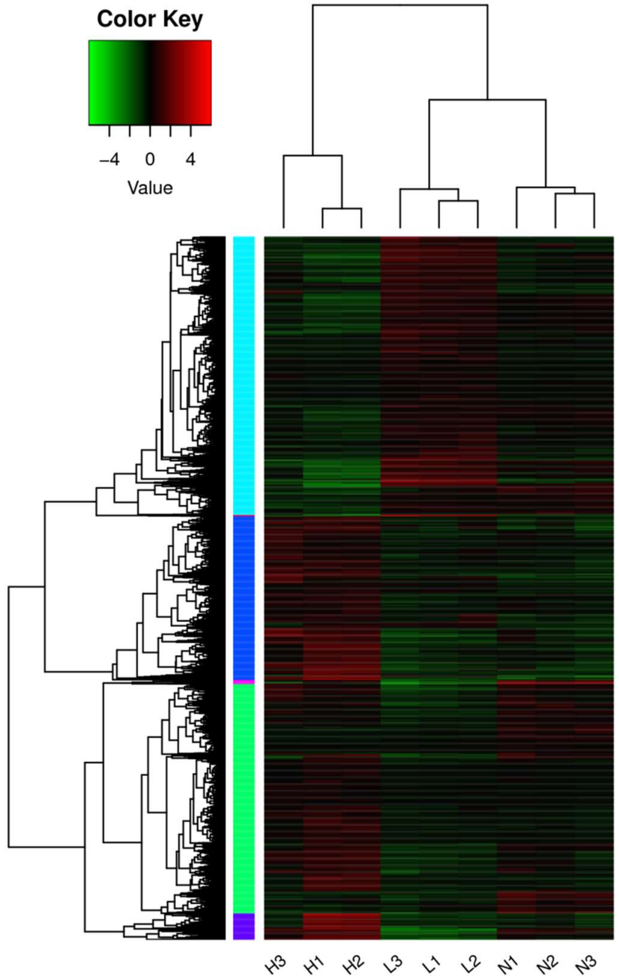

Fig.S5 HCA analysis of the DE genes from the three different groups of *Aurantiochytrium* sp. cells.

H: the high CSL-level group; N, the normal CSL-level group; L, the low CSL-level group.

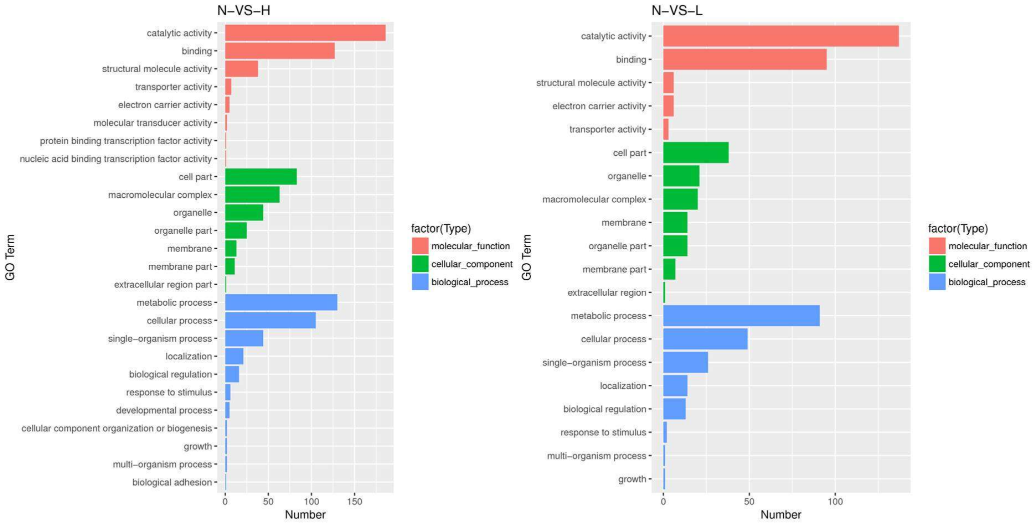

Fig.S6 GO classification for the DE genes from three groups of the *Aurantiochytrium* sp. cells.

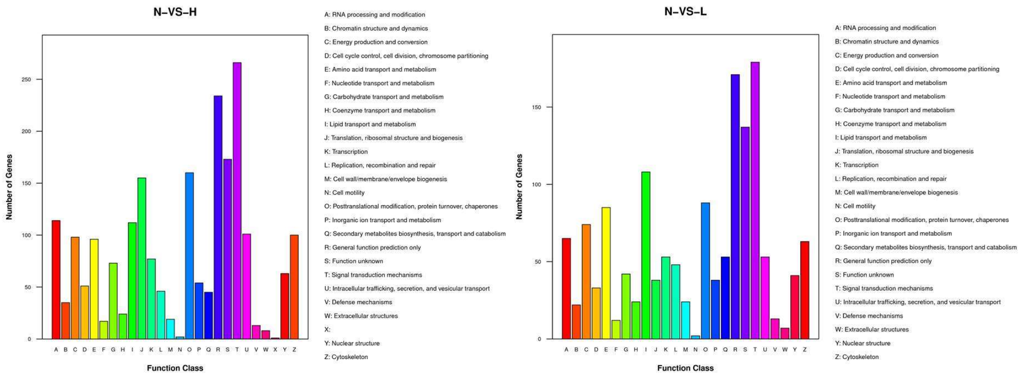

Fig.S7 COG categorization for the DE genes from three groups of *Aurantiochytrium* sp. cells.
